# Diagnosis and Treatment of Bone Metastases in Breast Cancer: Radiotherapy, Local Approach and Systemic Therapy in a Guide for Clinicians

**DOI:** 10.3390/cancers12092390

**Published:** 2020-08-24

**Authors:** Fabio Marazzi, Armando Orlandi, Stefania Manfrida, Valeria Masiello, Alba Di Leone, Mariangela Massaccesi, Francesca Moschella, Gianluca Franceschini, Emilio Bria, Maria Antonietta Gambacorta, Riccardo Masetti, Giampaolo Tortora, Vincenzo Valentini

**Affiliations:** 1 “A. Gemelli” IRCCS, UOC di Radioterapia Oncologica, Dipartimento di Diagnostica per Immagini, Radioterapia Oncologica ed Ematologia, Fondazione Policlinico Universitario, 00168 Roma, Italy; fabio.marazzi@policlinicogemelli.it (F.M.); stefania.manfrida@policlinicogemelli.it (S.M.); mariangela.massaccesi@policlinicogemelli.it (M.M.); mariaantonietta.gambacorta@policlinicogemelli.it (M.A.G.); vincenzo.valentini@policlinicogemelli.it (V.V.); 2 “A. Gemelli” IRCCS, UOC di Oncologia Medica, Dipartimento di Scienze Mediche e Chirurgiche, Fondazione Policlinico Universitario, 00168 Roma, Italy; armando.orlandi@policlinicogemelli.it (A.O.); emilio.bria@policlinicogemelli.it (E.B.); giampaolo.tortora@policlinicogemelli.it (G.T.); 3 “A. Gemelli” IRCCS, UOC di Chirurgia Senologica, Dipartimento di Scienze della Salute della Donna e del Bambino e di Sanità Pubblica, Fondazione Policlinico Universitario, 00168 Roma, Italy; alba.dileone@policlinicogemelli.it (A.D.L.); francesca.moschella@policlinicogemelli.it (F.M.); gianluca.franceschini@policlinicogemelli.it (G.F.); riccardo.masetti@policlinicogemelli.it (R.M.); 4 Istituto di Radiologia, Università Cattolica del Sacro Cuore, 00168 Roma, Italy

**Keywords:** bone metastasis, breast cancer, radiotherapy, diagnostic imaging, systemic therapies

## Abstract

The standard care for metastatic breast cancer (MBC) is systemic therapies with imbrication of focal treatment for symptoms. Recently, thanks to implementation of radiological and metabolic exams and development of new target therapies, oligometastatic and oligoprogressive settings are even more common—paving the way to a paradigm change of focal treatments role. In fact, according to immunophenotype, radiotherapy can be considered with radical intent in these settings of patients. The aim of this literature review is to analyze available clinical data on prognosis of bone metastases from breast cancer and benefits of available treatments for developing a practical guide for clinicians.

## 1. Introduction

Thanks to treatment implementations [1], metastatic breast cancer (MBC) has shown an improvement of outcomes in the last years. However, prognosis is still critical [2], with reported 27% 5-year survival rates [3]. Incidence of MBC interests 25–28% as de novo metastatic, while the rate of metastatic recurrence is reported in 20–30% of patients in western countries—and can be even higher in low- to medium-income countries [4]. Over time, the risk of becoming metastatic increases, and the data describe a cumulative risk of 4.8% (4.7–4.8) at one year, 5.6% (5.5–5.6) at two years, 6.9% (6.8–7.0) at five years and 8.4% (8.3–8.5) at ten years [5].

Bone metastasis commonly occurs in solid tumors; 36% of the incidence is from breast cancer [5], with a tendency of incidence in luminal subtypes [6]. In the surveillance epidemiology end result (SEER) database, a retrospective analysis based on subtype and incidence of distant metastasis, data on the first site of relapse show that bone metastases commonly involve luminal subtypes (ER+/HER2− 58.52% and in ER+/HER2+ subtype 47.28% of incidence) [6]. The ER−/HER2+ subtype has a higher proportion of liver metastases (31.72%), while the triple negative (TN) subtype is more affected by lung involvement (32.09%), with an incidence of bone metastases of 34.49% and 36.39%, respectively [6]. In a retrospective study by Molnar IA et al., the luminal A subtype presented a tendency of isolated bone metastases in 59% of cases [7]. In breast cancer, bone metastasis can occur in a de novo or recurrent setting, with a pluri- or oligometastatic presentation and may or may not be associated with other sites of involvement, so the spectrum of prognoses can differ greatly [6,8,9].

Etiopathology of bone metastasis is based on multicellular unit (osteoblasts, osteoclasts, bone lining cells, osteocytes) disruption with release of growing factors (TGF-B, FGF, PDGF, IGF) that promotes increase of tumor cell growth and compromise of secondary bone architecture [5,10]. In particular, biologic theory hypnotizes that, in sclerotic lesions, the tumor produces growth factors and induces osteoblast differentiation with the inhibition of bone resorption, while, in lytic lesions, tumor-derived factors enhance pro-osteoclastogenic differentiation and activity with consequently bone resorption [11].

Due to the release of chemical mediators, bone metastases are a common cause of cancer pain, with increasing of pressure in the bone, microfractures, stretching of the periosteum, reactive muscle spasm, nerve root infiltration, compression of the nerve due to collapse of the bone [12].

Skeletal-related events (SRE) are complications of bone metastasis growth and consist of pathologic fractures, spinal cord compressions and the necessity of radiotherapy for pain/impending fracture or surgery to bone. SRE can compromise performance status, with a reduction of quality of life, poor survival outcomes and also limited access to systemic therapies [13].

Thanks to new emerging diagnostic imaging and systemic therapies [14], alongside the most compromised presentations of bone metastases in breast cancer, we are assisting even more with oligometastatic presentations (de novo or inducted) [8]. Early detection of metastases—and possibly using targeting agents—can enhance disease control over time [2,15,16]. Associated with systemic therapeutic options, local treatments such as radiotherapy (RT), are possible options for the implementation of local controls—with both palliative and eradication intents [17,18]. The radiobiological aim of radiotherapy is to cause an interruption of the vicious biomolecular pain cycle with not only pain relief, but also decreasing the local tumor burden in more radiosensitive tumor subtypes [19]. It has been clinically demonstrated that patients obtain an immediate relief of symptoms in 2–4 weeks [11,20,21], and radiologically demonstrated that, for intent-to-eradicate treatments, local controls at 1 and 2 years can achieve 90.3% and 82.4% success with excellent safety [22]. For this reason, oligometastatic/oligoprogressive patients are even more challenging because physicians can imbricate local treatments such as radiotherapy with new systemic drugs to achieve higher progression-free survival—and in general, improve overall survival. In these settings, radiotherapy can also promote eradication of subclones resistant to systemic therapy.

Here we propose a review of diagnostic imaging for the early detection of bone metastasis in breast cancer, their use for radiotherapy targeting and local therapy options with a focus on radiotherapy possibilities in terms of dose and volumes and integration of chemoradiotherapy to improve clinical outcomes. The final purpose is to offer a practical guide for multidisciplinary management of patients with bone metastases from breast cancer.

## 2. Diagnostic Imaging for Bone Metastasis from Breast Cancer

The metastatic spread from a primary breast tumor can occur at an early, pre-symptomatic stage. Disseminated cells can lie dormant for years before becoming clinically evident [23]. In some studies [24,25], it has been shown that during the metastatic process of breast tumors, disseminated cancer cells at early stages of tumor evolution successfully establish themselves in the bone marrow [23]. Based on this theory, adjuvant systemic therapy (chemotherapy, target therapy and/or hormone therapy), is always administered when indicated.

In terms of correctly identifying subsetting and prognosis, it is challenging for physicians to precociously identify bone metastasis during staging and follow-up. Even more diagnostic and functional imaging are moving towards this goal. Today, with innovations in morphologic and functional exams, novel technologies offer possibilities to detect early bone metastases. Imaging is considered fundamental not only for diagnosis, but also as necessary to identify target lesions in local treatments. 

### 2.1. Morphologic Imaging

Morphologic exams, including radiographs or computed tomography (CT), are based on changes in bone density. Based on metastasis behavior, (lytic, sclerotic or mixed) metastases can present different pattern at imaging.

To be detected at CT exams, bone metastases need to be at least one cm with a loss of density around 25–50%. Usually breast cancer bone metastasis are lytic, but during treatments, due to response with osteoblastic reaction, they can become peripherally osteosclerotic. CT also allows to define soft-tissue invasion outside bone. Moreover, morphologic exams are fundamental to define critical site of bone metastasis which are at risk for SRE.

*Magnetic resonance imaging*. Conventional MRI sequences with T1, T2 and DWI studies, allow to detect breast cancer bone metastases with a sensitivity reported since to 100% [26] and a specificity of 90%, so they are used in case of doubt and are very useful for early detection. The pattern of MRI behavior of bone metastases usually determines low T1-signal, T2 hyperintensity and DWI signal restriction [27]. MRI allows visualizing lesions with high precision, and it is also useful to study integrity of spinal cord and eventually condition of its compression. For bone study, MRI is performed without contrast, but for study of spinal cord or surrounding soft tissue, contrast is required. Recently, whole-body MRI (WB-MRI) has been developed for study of entire bone compartment, but its utility for clinical practice is still under investigation—especially for early detection of bone metastasis [28]. In any case, its application could be interesting for early detection of oligometastatic patients. In literature, data on WB-MRI also provide a quantitative measure of treatment response in skeletal metastases and its sensitivity and specificity are superior to skeletal scintigraphy [29,30].

### 2.2. Functional Imaging

*Bone scintigraphy.* Functional imaging finds a role in staging, restaging and, during follow-up in detecting bone metastasis in breast cancer. The osteotropic agent used for skeletal imaging is metastable technetium 99 (99mTc) labeled diphosphonates for bone scintigraphy.

99mTc-radiolabeled diphosphonates has been in use since 1970s and thanks to its effectiveness and low cost, it is worldwide dedicated to first-level staging. Reported sensitivity and specificity are 78 and 48%, respectively [27,31]. Bone scintigraphy usually detects bone turnover, so metastasis with a prevalent lytic behavior can be considered as false negative. An alteration, not exclusively cancer-related, in 5–10% bone can cause accumulation of agents on bone scans, though this can be also a confounding factor with a benign pathology such as degenerative disease. For this reason, a second-level exam can be required in borderline cases. Another limitation of bone scan is represented from absence of volumetric evaluation and poor spatial resolution (<1 cm). An implementation of a bone scan is represented by single photon emission CT (SPECT/CT), in which the same radionuclide used for conventional skeletal scintigraphy is injected during acquisition of additional axial slices, with the possibility to have volumetric evaluation.

*Positron emission tomography (PET)*. PET is superior to the bone scan in terms of spatial resolution with acquisition of tomographic images. It also provides information about treatment response and prognosis [32]. Most employed radiopharmaceutical agents for skeletal investigation are 18F labeled sodium fluoride (18F NaF) and 18F labeled fluorodeoxyglucose (18F FDG). Due to fluoride ions collocation in the remodeling skeletal areas, 18F-NaF PET is particularly sensible to osteoblastic activity. 18F-NaF PET presents a high sensibility (100%) and specificity of 97% and it is more efficacy to detect bone metastases than 18F FDG, though it is still to be defined the setting of patients in which it could be useful [27]. About breast cancer, indolent subtypes with bone tropism such as luminal or lobular cancer, could be considered for specific protocols with 18F-NaF PET. Moreover, these subtypes with slower cellular growth and consequent lower uptake of glucose, present a poor sensibility of 18-FDG PET/CT and their spread could be missed.

18-FDG PET/CT is instead considered useful in case of locally advanced or metastatic disease for staging, evaluate treatment response and prognosis [33]. Accumulation of its agent is in high turnover areas. The sensitivity and specificity of 18F-FDG PET for detection of bone metastasis is 98% and 56%, respectively, even if it can be different according to subtypes [27]. Indication for use in follow-up is still controversial.

*Hybrid images*. A recent review by Cook G et al. [29] reported that molecular and hybrid imaging has an increasing role in early detecting of bone metastases and in monitoring response at early time points. In this sense, functional imaging as emission computed tomography (SPECT/CT), positron emission tomography/CT (PET/CT) or PET/MRI in breast cancer could find a role in identified early patients not responder to systemic therapies for shifting to further line of treatment with a benefit on disease control and cost/effectiveness of health systems. This advantage is based on combination of morphologic, physiologic and metabolic aspect for skeletal evaluation. Comparing the data in literature, the advantages of PET/MRI are still few and studies are focused on finding the best setting of patients [34].

### 2.3. Diagnostic Imaging for Treatment Planning of Radiotherapy

Morphologic imaging is useful for identify bone lesions and soft tissue invasion. In palliative radiotherapy treatments of bulky metastases, CT scan simulation allows radiotherapist contouring also of soft tissue surrounding. In some cases, co-registration with diagnostic CT scan with contrast can be helpful for distinguish healthy soft tissue from that interested by spread of disease outside bone metastases. MRI is useful for treatments with radical intent because it allows higher precision in gross tumor volume (GTV) and spinal cord contouring. Increased accuracy is always associated with higher local control and less side effects. MRI is usually required for stereotactic body radiotherapy (SBRT), in which target of the treatment is the lesion with a millimetric margin and dose are high. Functional imaging is less strictly used for contouring of bone metastasis in breast cancer and hold a function of supporting detecting of lesion at co-registration.

### 2.4. Biopsy on Bone Metastasis: When Imaging Is Not Enough

Metastatic presentation—especially in case of relapse—usually required a biopsy for prognostic factors study to confirm nature of disease and setting of systemic therapies. More often, in case of de novo metastatic patients, soft-tissue or primary tumor undergo pathologic study, while in case of relapse, especially for isolated bone presentation, a biopsy of lesion can become mandatory. Other conditions in which biopsy can be mandatory are necessities of differential diagnosis. The differential diagnosis for bone metastases includes chondrosarcoma, primary malignant lymphoma of the bone, multiple myeloma, post-radiation sarcoma and osteomyelitis. A distinction between acute osteoporotic fractures versus metastatic fractures should be made on radiographic imaging. In osteoporosis, the cortical bone may appear preserved, while in secondary lesions, cortical bone is typically destructed. Another possible differential diagnosis is sarcoidosis, because lesions cannot be reliably distinguished from metastatic lesions on routine MRI studies [35]. 18F-FDG PET/CT is highly sensitive in detecting granulomatous bone marrow infiltration, but an increased 18F-FDG uptake can mimic metastatic disease, reducing the specificity of 18-FDG PET/CT when both sarcoidosis and a tumor which may develop bone metastases occur in the same patient [36].

## 3. Radiotherapy Treatments Options and New Drugs

**Figure 1:**
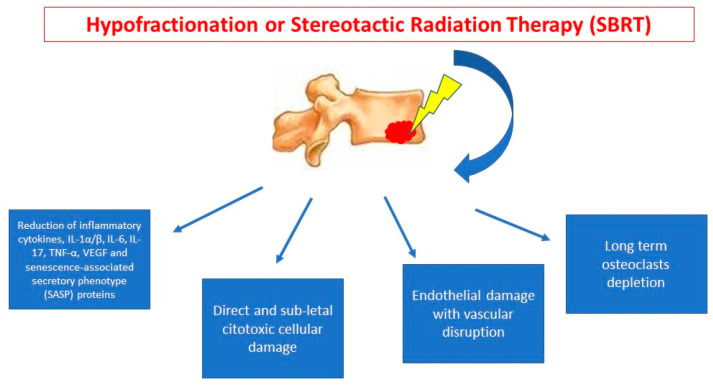
Radiotherapy tissue damage mechanisms in bone metastases.

*Radiotherapy effect on bone metastasis.* In-human pathologic data of radiotherapy damage on bone metastases are few. In general, RT effect is mediated by sublethal damage from free radical generated by water molecules or, in case of high doses, also direct lethal damage on DNA [37]. In fact, higher doses for fraction, as in stereotactic radiotherapy (SBRT), can promote direct cytotoxic, endothelial disruption with vascular death [38,39] (Figure 1). On bone metastases, final effect of RT damage is reduction of pain (by interruption of biomolecular pain modulation mechanisms), interruption of osteolysis mechanisms and decrease of tumor burden [40]. Radiotherapy with palliative intent causes an interruption on neuromodulatory algic mechanism by early depletion of inflammatory cells, thanks to inhibition of the inflammatory cells [12]. Main trigger of pain modulation by bone metastases are nerve growth factor (NGF), bradykinin, serotonin, adenosine triphosphate, H+, lipids (prostaglandin E2) and degenerin family of ion channels [12].

Decrease of osteolysis is mediated by osteoclasts apoptosis, as in vitro data showed [41]. Radiotherapy can also promote reossification process from 3–6 weeks from the end of radiotherapy and reaches highest degree within six months [11]; ossification process is realized in 65% to 85% of lytic metastases in unfractured bone [12].

In a study by Steverink et al. [42], on ten biopsy of vertebral metastasis who underwent a single preoperative SBRT of 18 Gy, a change of tissue in 21 h, as necrosis development, happened in 83% of sample. A consistent reduction of mitotic activity and vessel density (especially in renal cell metastases who are enriched of vessels) was also reported. On these samples, pathologic analysis underlined a persistence of T-cell and natural kill cell density after SBRT. Probably, in a further phase, immune-related reactions starts against antigens exposed by tumor cell damage.

From radiobiological data on primary tumor, in which lesions since to four centimeters were treated with definitive radiotherapy, 3-year local control of 81 and 100% were achieved with doses of 70–80 Gy and >80 Gy, respectively [43]. Some authors speculate that a large single fraction could be more advantageous on breast cancer, compared with prolonged fractionated radiotherapy [44]. For the tissue damage caused, radiotherapy can be considered crucial as local ablative treatment in oligometastatic breast cancer setting especially when a BED > 75 Gy [45].

*Dose and volumes of radiotherapy treatments.* Dose and volume prescriptions are chosen according to aim of treatment. In palliative setting, radiotherapy aims to control symptoms and local growing of disease. It is usually combined with antalgic drugs modulation and orthopedic multidisciplinary evaluation can be required for setup and mobilizing patients during RT. Palliative RT volumes usually include all the bone compartment and extra compartment invasion of lesion, with sub-centimetric margins. Historically, these treatments are administered with 3D conformal treatment plan with one or more fields of therapy, but at the present day, especially in case of retreatment, even more sophisticated techniques such as intensity modulated radiotherapy (IMRT) or volumetric modulated arch therapy (VMAT) can be chose for optimizing dose distribution, avoiding missing target and preserving organ at risk, especially spinal cord. Palliative radiotherapy is brief with administration of 8–20 Gy in 1–5 daily fractions (Fr), to obtain a pain relief or, in some cases, control of neurological impairment in some weeks [20,21] (Table 1a). In a metanalysis of Chow E et al. it is reported that efficacy of single-fraction RT and multi-fractions (since to 30 Gy in 10 Fr) are equivalent in terms of pain control, but rate of retreatment are 2.5-fold higher in single-fraction arms [46]. Patients who underwent surgery for SRE can benefit of adjuvant radiotherapy on surgery bed and residual disease. A prospective study on bone metastases with spinal cord compression showed that responsiveness of breast cancer tumor (that presents intermediate radiosensitivity) is linked to schedule of 30 Gy given with 10 Fr, while dose escalation is not related to an improvement of outcomes [47].

In oligometastatic settings, treatments with radical purpose are usually given in few days, but total doses reach a higher biologic equivalent dose (BED), of at least 75 Gy [45,48,49] (Table 1b). For these treatments, higher sophisticated techniques are usually used to conform volumes and stereotactic body radiotherapy technique (SBRT) is often applied for sparing organ at risk and give higher doses on the core of GTV. SBRT requires strictly system of immobilization and co-registration with MRI is mandatory to detect bone lesion and for spinal cord identification [50].

In literature, few retrospective and prospective series reported data on oligometastatic breast cancer, but results show that a treatment direct to metastases (surgery or radiotherapy) is significantly related to survival outcomes at 10–20 years [17,18]. Patients who are candidate to these treatments need to be carefully selected in terms of prognosis [55]. In general, breast cancer is a favorable prognostic factor for OS in oligometastatic patients who underwent SBRT (HR, 0.12; 95% CI, 0.07–0.37) [56]. Another prognostic factors that has been found related to OS in a retrospective SBRT for oligometastatic analysis was BED > 75 Gy [48]. In a study by Milano MT et al. survival outcomes of SBRT in 48 oligometastatic breast cancer treated for extracranial metastases showed that bone-only oligometastatic present a younger age, usually are hormone-responders and synchronous with diagnosis [17]. In this study, OS and freedom from widespread metastases (FFWM) were better in bone-only group (12 patients); these patients underwent RT with a median EQD2 of 57.3 Gy [38.3–70]. In a Phase II prospective trials, oligometastatic breast cancer patients were treated on all metastatic sites with SBRT (30–45 Gy in 3 Fr) or IMRT (60 Gy in 25 Fr). Results showed that 60 on 92 metastatic lesions were in the bone and 80% of patients included were Luminal A. In this study, 1- and 2-year PFS was 75% and 53%, respectively; two-year LC and OS were 97% and 95%, respectively, while only one bone lesion on 60 relapsed in spine (but was treated with 17 Gy in 3 Fr) [52].

In another study of 2015 by Yoo GS et al. 50 patients with bone metastases who underwent RT for a median dose of 30 Gy (20–60 Gy) were retrospectively studied. The analysis of Yoo GS showed that patients treated with a BED of at least 50 Gy presented better 5-year LC and OS [51]. In a prospective cohort of 50 patients with breast cancer, 68 spine bone metastasis were treated with a single fraction radiosurgery for a total mean dose of 19 Gy (15–22.5 Gy) with a 96% of pain control and local control at 15 months of 100% [44]. In a mixed cohort of 22 oligometastatic and oligoprogressive patients, 32% were affected from breast cancer and were treated with doses from 35 to 50 Gy in 5 Fr, to spinal and non-spinal metastases, respectively [53]. Local control achieved was 91% at 1-year, with median PFS and OS, respectively of 10.1 and 37.3 months, while PFS stratified for OP and OM group were 6.6 and 10.6 months, respectively.

Some patients are not candidate to stereotactic radiosurgery (SRS), for presence of more than three lesions or for proximity to spinal canal and an intermediate solution to achieve a better local control is to administer a simultaneous integrated boost (SIB) on GTV, treating whole vertebra with palliative dose and fractionation. In a cohort of 12 patients, of which only one was affected by breast angiosarcoma (with a different radiosensitive respect breast carcinoma), treatment with a SIB of 40 Gy and 30 Gy on whole vertebra given in 10 Fr showed a 1-year LC of 93% [54].

At the present time, there is a great inhomogeneity in dose prescription especially for extraspinal bone metastasis and the need of consensus guidelines supported by evidences is necessary [57,58].

*Cytotoxic chemotherapy and radiotherapy*. Systemic therapy is still the fundamental treatment for all molecular subtypes in the management of MBC with bone metastases [33,59]. Drug choice is influenced by immunophenotype, previous treatment and tumor spread [33,59]. In TNBC or hormone-resistance MBC anthracycline- or taxane-based regimens are preferred treatment [60,61]. Recently, therapeutic options after anthracycline- and in case of taxane-resistant disease were increased. In fact, some cytotoxic drugs after first line chemotherapy treatment are become available. In the last years, eribulin [62] and nanoparticle albumin-bound paclitaxel [63], in monotherapies administration, were added to therapeutic options that have long been available as capecitabine, vinorelbine, cyclophosphamide, gemcitabine and pegylated liposomal doxorubicin [64,65]. In addition, combination therapies such as paclitaxel plus gemcitabine or carboplatin plus gemcitabine could represent an alternative option, but sequential monotherapy is usually preferable in MBC setting [62,63]. Generally, bone metastases had the low response rates to chemotherapy. For this reason and for the need of a rapid pain relief, these systemic treatments are often imbricated with palliative radiant treatment. In oligometastatic setting, to imbricate radiant treatments with cytotoxic treatment it can be considered to achieve a better disease control, discussing case by case. In both cases, considering the significant risk of myelosuppression of both treatments, radiotherapy is almost never concomitant with systemic treatment. The clinicians must merge these treatments to avoid the overlap of the specific nadirs of bone marrow toxicity. The sequence of these treatments is dictated by the need to prioritize a systemic control of disease versus a locoregional control (oligoprogressive) or the pain control.

*Hormonal therapy and radiotherapy.* In MBC patients, bone metastases more often derived from HR-positive tumors as previously described [7]. In this case hormonal therapy (ET) is the preferred choice in most cases, except for rapidly progressive disease or in case of visceral crisis, where cytotoxic drugs remain the preferred option [59]. In recent years, the introduction of everolimus (M-TOR inhibitor) [66] and alpelisib (PI3KCA inhibitor) [67] in hormone refractory disease and CDK4/6 inhibitors [14,68] in both hormone-sensitive and hormone-refractory disease has made hormonal sequence more complex and often longer.

*Target therapy in ER+HER2− setting and radiotherapy.* Recently, target therapies became even more common in ER+/HER− metastatic setting. In addition, frequent presence of bone metastases has also determined the need to imbricate these systemic therapies with palliative or radical RT. Hormonal treatment alone, characterized by an excellent toxicity profile, not arises problem for combination with radiotherapy, association with target therapy instead entails timing issue for imbrication. No prospective studies, addressed to establish the best combination schedule between target therapy and RT, are currently ongoing. Continuous and semicontinuous therapeutic schedules for these target therapy, implying necessity of treatment discontinuation in case of necessity to decrease cumulative toxicity. As regards everolimus and alpelisib, in the absence of clinical data, no biologic contraindications can be postulated at the basis of the need for drug suspension during radiant treatment. Vice versa, for using of CDK4/6 inhibitors (ribociclib, palbociclib or abemaciclib) that act directly on the cell cycle, it is evident that optimization of the combination with radiant treatments appears to be a goal to be achieved. In pivotal studies of CDK4/6 inhibitors, radiotherapy is allowed before systemic therapies beginning and it is preferable to avoid concomitance [68,69,70]. In literature, few data are reported that showed feasibility of radiotherapy in concomitance with CDK4/6 inhibitors, with a possible side effects arising (for example there are some reports of GI toxicity with RT on bone metastasis during abemaciclib) [71,72,73,74]. Another interesting issue about radiotherapy and CDK4/6 inhibitor is time of association because these drugs cause a cell blockage in G1 phase with consequently possible radioresistance. At the end, hypothetic effect on immune system by CDK4/6 inhibitor could be implemented with ablative RT, and it is under investigation in Phase II protocol ongoing.

*HER2 target therapy and radiotherapy.* In preclinical studies in vivo and in vitro, it is clearly identified that HER2−overexpression is a factor of radioresistance in breast cancer [75,76,77,78]. It seems that the PI3-K/Akt pathway, increase of antiapoptotic transcription factors (NF-KB and c-myc), Fak protein [79] or STAT3-survivin signaling [80] are implicated in the mechanisms of radioresistance of HER2 positives breast tumors.

Recently, in clinical practice, since from trastuzumab (the first anti-HER2 monoclonal antibody) many treatments have been developed that are revolutionizing the systemic therapy of HER2 positive disease. Various anti-HER2 TKIs such as lapatinib, neratinib and tucatinib and new anti-HER2 monoclonal antibodies such as pertuzumab and T-DM1 have been introduced in recent years.

Concerning the trastuzumab, some authors [81,82] described HER2−dependent sensitization to radiation-induced apoptosis by trastuzumab in a panel of breast cancer cell lines. This radiosensitizing effect was not associated with toxicities as demonstrated in preclinical model [83]. Furthermore, in MBC patients, concomitant administration of trastuzumab with radiotherapy does not increase major toxicity, particularly cardiac.

Moreover, for lapatinib and T-DM1, there are preclinical study with xenograft of HER2−positive breast cancer cells where the radiosensitizing effect of these drugs is confirmed [84,85,86]. Even in small clinical trials lapatinib and T-DM1 given at standard dose (respectively 1500 mg/day per os and 3.6 mg/kg intravenously every three weeks) in combination with RT were well tolerated [87,88]. However, it is important to note that a significant number of cases of radionecrosis was reported with concomitant T-DM1 and SRS for brain metastases in HER2−positive MBC.

Overall, the available data show a good efficacy profile and poor toxicity for the combinations between anti-HER2 therapy and radiotherapy, however these data often concern small numbers of patients, many are retrospective or do not directly compare the concomitant association.

*PARP inhibitors and radiotherapy.* Poly(ADP-ribose) polymerase (PARP) proteins catalyze the polymerization of poly(ADP-ribose) on proteins. This reversible post-translational modification of proteins—also called parylation—has been implicated in many cellular mechanisms, notably DNA repair. PARP detects single-strand breaks (SSBs) and—through its parylation activity—recruits proteins that mediate DNA repair such as XRCC1, which stabilize the DNA break. DNA polymerase performs complementary base synthesis, and DNA ligase III ligates the ends of the DNA [89]. Ultimately, the auto-parylation of PARP releases it from the SSB site. PARP activity is enhanced in many tumors [90]. Thus, the inhibition of PARP activity is being used increasingly as a therapeutic strategy especially in MBC with BRCA mutations. Two PARPis are recently showed an interesting efficacy in BRCA mt MBC (olaparib and talazoparib). Radiosensitizer molecules are used to enhance the effects of radiation on tumors, improving the antitumor response with lower toxicity. PARPis are potential radiosensitizers, based on their ability to enrich unrepaired DNA damage [91]. In tumor models comprehending breast cancer, PARPis have had good efficacy as radiosensitizers, with an enhanced of cellular death. Their effects included inhibition of tumor cell proliferation, decreased cellular survival, delayed tumor growth and improved survival in mice [92]. However, the radiosensitizing effect of this combination raises concerns about its toxicity, the secondary hematological effects of PARPis, such as myelosuppression [93], could amplify when combined with pelvic or large-field spinal radiation. Taken together these consideration, the rationale for the concomitant use of PARPi and radiotherapy is strong, however, in light of the bone marrow toxicity profile, in the absence of prospective trials with verified dosage of the drug, we do not recommend the concomitant use of these treatment with radiotherapy.

*Immunotherapy and radiotherapy*. Although immunotherapy has shown antitumor activity against several advanced tumors in recent years, at the present day for breast cancer data showed in TN promising results. In fact, atezolizumab plus nab-paclitaxel in PD-L1 positive metastatic TN population has shown an increase of PFS and OS respect chemotherapy alone [94]. The spread of bone metastases activates many immunosuppressive pathways. Therefore, the immunophenotype of bone metastases could represent a different pattern of response to immunotherapy when compared to visceral disease. Though checkpoint inhibitors have shown significant efficacy in many tumors including TN breast cancer with visceral metastases, their specific performance in bone metastases is not well understood and it may be poor. Although we currently have not clinical data, radiotherapy on bone metastases could make these localizations of disease more immunogenic and optimize the effectiveness of inhibitory checkpoints. Given these considerations, studying how and when to combine these treatments is an important goal of clinical research in the coming years.

*Further perspectives.* At the present time there is an increasing interest in oligometastatic breast cancer, especially in good prognosis setting (Luminal subtype, single lesion, bone metastasis only). Ongoing trials are investigating possible therapeutic patterns in this sense. In April 2020, a Medline on ClinicalTrial.gov showed that six trials were active for oligometastatic while only one trial was active for oligo progression.

A Phase II trial (CLEAR, NCT03750396 in Table 2) is dedicated to oligometastatic recurrent patients (all parenchyma) with ER+/HER2− who underwent a radical local approach [surgery, radiotherapy (57–97.5 Gy/6–10 Fraction) or radiofrequency] during first systemic line to test PFS. Another trial (NCT02364557) is recruiting patients with limited MBC, randomizing them between systemic therapies (according to standard of care) and systemic therapies with association of stereotactic radiosurgery in one, three or five fractions at the discretion of the treating physician, to test PFS and OS. A Phase III study, STEREO-SEIN Trial, (NCT02089100) is testing the role of curative SBRT in de novo oligometastatic breast cancer (no triple negative subtypes), randomizing patients between systemic therapies (according to standard of care) and systemic therapies with association of stereotactic radiosurgery. In another trial (NCT03808337), supported by Memorian Sloan Kettering Cancer Center, is recruiting metastatic non-small cell lung cancer or triple negative breast cancer, with randomization between standard systemic therapies vs. receiving SBRT (with a minimum BED more than or equal to 48 Gy_10_) to all sites of metastasis, concurrently with systemic therapies. In another Phase I/II trial by NCI (NCT00182793), patients with Stage IV Metastatic and Stage IIIB/C Breast Cancer were enrolled to receive bone marrow ablation with chemotherapy and autologous-autologous tandem hematopoietic stem cell transplantation and concurrent RT on site of disease. In this study, oligometastatic patients, received helical-tomotherapy RT on site of metastases with standard fractionation. In CIMER study (NCT04220476), a Phase II study, patients with oligometastatic BC, luminal subtypes, who are candidates to first-line with CDK4/6 inhibitors will be randomizing between receiving first-line of treatment vs. underwent also immune-SBRT every 48 h on all sites of metastases with a total dose of 50 Gy in 5 Fr.

At the present time only one trial (NCT03808662) is testing oligoprogressive setting in NSCLS and TNBC patients, randomizing them between standard of care and SBRT 9–10 Gy × 3 or 10 Gy × 5 fractions given every other day to all oligoprogressive sites.

## 4. Co-Adjuvant Systemic Therapies and Focal Alternatives to Radiotherapy

*Bone target agents.* To control skeletal disease, some other focal therapies have been developed and used in clinical practice, such as systemic therapeutic agents. First, bone target therapies which are systemic agents used to control skeletal disease frailty, even if in case of bone metastases. Behind oncological systemic therapies for breast cancer, two main groups are available, antiresorptive drugs and bone-seeking radiopharmaceuticals. Antiresorptive drugs aim to control both bone metastases incidence in adjuvant setting and their advantage in breast cancer is well consolidated [11]. Bisphosphonates and denosumab are commonly used in clinical practice and their therapeutic effect is based on targeting locoregional tissue cells to activate not only blocking of resorption mechanism, but also activating antitumor response by immune system activation.

*Radiopharmaceuticals.* Radiopharmaceuticals drugs are principally used for pain relief in palliative setting with involvement of more than one skeletal site [95]. Therapeutic bone-seeking radiopharmaceuticals can be divided into two principal chemical classes: cationic or calcium-analog (phosphorus-32, strontium-89 chloride and radium-223 chloride) which are incorporated as calcium in bone regions thank to mineralization process and anionic or noncalcium-analog (Samarium-153 lexidronam and rhenium-186 etidronate) bone-seekers with different mechanism of uptake into bone by chelating mechanism to organic phosphates. In literature, few experiences are reported on breast cancer patients. First of all, experiences with strontium-89 chloride (89 Sr) showed 75% of pain relief at two-three weeks from end of treatment [96]. Some other series reported results of use of rhenium-186 etidronate (186 Re–HEDP) on metastatic breast cancer patients with implementation of quality of life of 58% and pain relief of 60% [97,98]. To preserve bone marrow function, recent develop of alfa-emitter that present a short radiation range, has been applied also to metastatic breast cancer. radium-223 chloride (223 Ra) was administered in a Phase I study on 10 breast cancer metastatic patients with results of a pain relief since to 60% and absence of G3 bone marrow events [99]. In another study, breast cancer patients with predominant bone disease underwent 223 Ra therapy with metabolic activity reduction of lesions and a good safety profile [100]. CARBON trial, registered o 2016, is investigating a possible combination of 223 Ra and capecitabine in terms of safety and disease control for metastatic breast cancer patients with bone involvement [101]. Limitations of radiopharmaceuticals is their myelosuppressive persistent effect and indication to use principally in the palliative setting. No data are in favor of their use in preventive or oligometastatic setting in absence of symptoms.

*Surgery*. Bone metastases cause an impairment in bone density and architecture that has a negative impact on mechanical performance of bone, especially for support and motorial function [102]. Surgery can be considered both for excisional and palliative intention. Excisional surgery includes wide procedures, hemipelvectomy, wide resection with prosthesis, curettage and cementing, while palliative surgery includes internal and external fixation [103]. Although bone is only one possible site of metastatic lesions and local control on bone metastatic sites has a little effect on global status of disease, excisional intention of surgery can be considered in case of confined disease (oligometastatic, one parenchyma involved), to improve quality of live [103]. Surgery needs also healing time respect other therapies for local control and its indication needs to consider also systemic therapies ongoing and their time of suspension. Many target therapies for metastatic breast cancer can require a stop for side effects in terms of bone marrow suppression, to avoid post-surgery complications. Moreover, some drugs are cytostatic, and this can increase time for healing. Delaying systemic therapy in oligometastatic patients can reduce global disease control. A proper algorithm for establishing a diagnosis and evaluation of prognostic factors would help in planning the surgical intervention. In a study of Durr et al. a series of 70 patients with breast cancer bone metastases were treated with surgery and of the 19 patients with solitary bone lesions, only 26.3% (5 patients) were alive and free of disease at a mean follow-up of 35 months [104]. This retrospective study found that only two independent factors for survival were extent of disease and duration of symptoms from bone lesions, so they concluded that orthopedic surgery in patients with bone metastases secondary to breast cancer, wide resection is not likely to be necessary [104]. In another study by Szendroi et al. an algorithm based on staging, prognostic factors and patients’ condition for classification and surgical treatment of bone metastases was proposed. Patients with solitary metastasis and good prognostic factors can be considered for surgery with radical intent or minimal surgery (palliative) followed by radiotherapy, while patients with multiple metastases are candidate in case of impending fractures to palliative surgery, if global conditions are acceptable [105].

Palliative surgery usually is required for fracture or risk of fracture and/or neurological vertebrae symptoms in patients that present a systemic compromising with a prognosis of at least 6–8 weeks, to implement quality of life.

*Interventional Radiology.* Interventional radiology includes different therapeutic techniques all with the aim of stabilization of the bone and improvement in quality of life [106]. Percutaneous techniques include vertebroplasty that allow to inject surgical cement in the vertebral body with immediate and analgesic effect in few days—and more recently—cementoplasty, that stabilize also of extraspinal lesion, for example long bone sites. Other percutaneous technique, such as embolization (with pure alcohol and contrast), radiofrequency (with a hot needle since to 65 °C) and cryoablation (with a generation of temperature −100 °C) can also cause tumoral cell destruction and need to be carefully used in case of proximity with nerve and vascular structures. Endovascular techniques cause a loss of blood flow inside bone lesions and this can reduce pain by reducing of pain modulators circulation. In case of big masses these techniques reduce systemic reaction of cytokines release. Endovascular techniques are embolization that uses microparticles or liquid agents and chemoembolization that uses antimitotic drugs (adriamycin and platinum derivatives) with also antitumoral effect.

Respect surgery procedures, interventional radiology present rapid healing time, but further prospective studies need to test their application in specific sub settings of metastatic breast cancer patients.

## 5. Implication for Clinicians

According to time, disease presentation and prognosis, bone metastases from breast cancer patients can be addressed to different pathways of care, for optimize symptoms management and outcomes. It is mandatory to identify patients who are at risk to develop bone metastases to tailoring diagnostic exams and therapeutic intervention. In a study by Colleoni et al. the highest cumulative incidences of bone metastases at any time were among patients who had four or more involved axillary nodes at the time of diagnosis (14.9% at two years and 40.8% at 10 years) and among patients who had as their first event a local or regional recurrence or a recurrence in soft tissue, without any other overt metastases (21.1% at two years from first recurrence and 36.7% at 10 years) [107]. Hence, it is important to tailor follow-up in patients that can be considered at high risk of relapse.

The therapeutic pathway can be tailored for each patient, and often requires multidisciplinary interventions, since from individuation of patients at risk already during follow-up. Negative prognostic factors for developing bone metastases reported in literature are: tumor size (>5 cm), higher tumor grade, tumor subtypes (lobular carcinoma), number of positive lymph nodes, extent of disease, duration of the symptoms, age > 60 years and hemoglobin less than 11 g/L, while positive prognostic factors found were estrogen receptor positivity, solitary bone presentation, bisphosphonate treatment.

Based on this literature review, we summed up all available results in an algorithm for practical use. The algorithm begins with identification of presence of metastases. This is fundamental, both at staging and follow-up patients should be investigated with tailored approach and studied with diagnostic exam according to their risk of metastases development and symptoms.

At confirmation of metastases, according to literature results and in consideration of the need to classify the type of bone metastasis presentations to optimize the treatment, we can divide them, in the sequent subgroups:

- De novo or recurrent metastatic breast cancer: based on time of metastases presentation;
- Oligometastatic or plurimetastatic breast cancer: based on the presence of five metastatic sites or more;
- Bone-only or visceral metastatic breast cancer: based on parenchymal involvement.

After qualitative and quantitative definition of metastatic disease, patients need to be stratified in prognostic group to chose best therapeutic options. In literature are reported as prognostic factors, age, ECOCG, comorbidities, immunophenotype, previous treatments. In fact, a TN old patient with isolated bone metastases will have a different prognosis of a luminal A plurimetastatic young patients.

**Figure 2:**
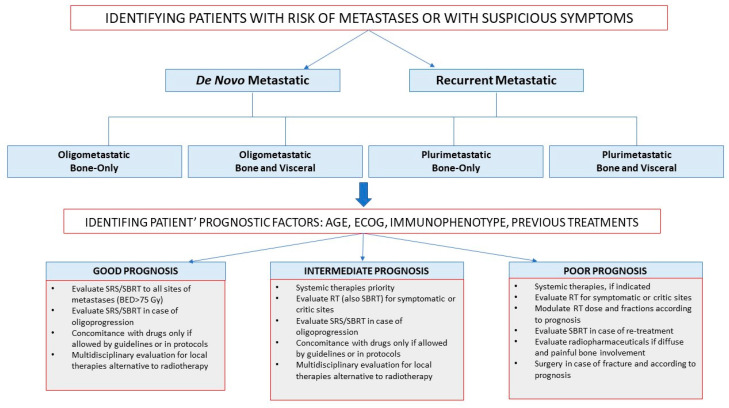
Therapeutic algorithms approach to patients with Bone metastases from breast cancer according to good, intermediate or poor prognosis.

According to prognostic subgroups’ organization, here we report an algorithm pathway for radical and palliative setting management of these patients (Figure 2). In this algorithm, patients with good prognosis are candidate to insertion of radical local therapies with definitive intent (radical radiotherapy SBRT/SRS, intervention radiology, surgery) on bone metastases during systemic therapies, but concomitance with drugs it is still under investigation (for achieve a better disease control). Patients with intermediate prognosis are the most heterogeneous group so for their management it is important to considered also prognostic factors (ER expression, age, performance status). They are addressed principally to maintain their systemic therapies and local treatments are introduced in case of symptoms and not compromised systemic situation. Patients with poor prognosis are candidate to therapies (systemic or local) that have the purpose to preserve quality of life, so also treatment of their bone lesions with radiotherapy or other techniques is considered with this intent.

## 6. Conclusions

Bone metastasis is a condition that unfortunately still affects patients with breast cancer, also limiting quality of life. Among these patients, oligometastatic breast cancer with only bone presentation represent a subgroup with favorable prognosis and in which escalation of diagnostic imaging methods, systemic therapies and imbrication with SBRT can be related with survival. Use of few or single-fraction SBRT can allow physician to administered BED of 75 Gy and to treat, with a radical intent, patient who present good prognosis.

Despite the considerations that can be drawn from currently available data, large pooled analysis and prospective trials are required to individuate best therapeutic algorithms, also considering new target therapies and the need of imbrication these treatment with radiotherapy to improve QoL and survival of our patients.

## Figures and Tables

**Table 1:** Radiotherapy dose and volumes for bone metastasis treatment.

**(a) Dose and Volumes for Palliative Radiotherapy on Bone Metastasis**			
**Dose**	**Volume**	**Outcome**	**Reference**
8 Gy 1 Fr	Bone compartment +/− soft-tissue invasion	Symptom control (pain, neurological impairment) Preferable in case of poor expectation of retreatments	Chow E. 2002 [20] Chow E. 2007 [46] Chow E. 2012 [21]
20 Gy 5 Fr	Bone compartment +/− soft-tissue invasion	Symptom control (pain, neurological impairment)	Chow E. 2002 [20] Chow E. 2007 [46] Chow E. 2012 [21]
30 Gy 10 Fr	Bone compartment +/− soft-tissue invasion	Symptom control (pain, neurological impairment) After surgical stabilization	Rades D, 2004 [47]
**(b) Dose and Volumes for Radical Radiotherapy on Bone Metastasis**			
**Dose**	**Volume**	**Outcome**	**Reference**
EQD2 of 57.3 Gy [38.3–70] BED 60 Gy (obtained)	Bone lesion + margin (mm)	5-year OS 83% (BO vs. no-BO *p* = 0.002) 10-year OS 75% (BO vs. no-BO *p* = 0.002) FFWM (BO vs. no-BO *p* 0.018)	Milano MT, 2019 [17]
BED > 50 Gy	Bone lesion + margin (mm)	3-year DPFS 36.8% 5-year LC 66.1% 5-year OS 49% univariate Analysis: Higher RT dose (*p* = 0.002) Whole Lesion RT (*p* = 0.007)	Yoo GS, 2015 [51]
30–45 Gy 3 Fr	Bone lesion + margin (mm)	1-year PFS 75% 2-year PFS 53% 2-year LC 97% 2-year OS 95%	Trovò M, 2017 [52]
15–22.5 Gy 1 Fr	Bone lesion + margin (mm)	15-month pain control 96%	Gerszten PC, 2005 [44]
35 Gy (spinal) 50 Gy (no spinal) 5 Fr	Bone lesion + margin (mm)	1-year LC 91.2% PFS 10.1 months OS 37.3 months	Kam TY, 2019 [53]
40 Gy (GTV) 30 Gy (WV) 10 Fr	Bone lesion + margin (mm) Whole vertebra (WV)	1-year LC 93% OS 58%	Farooqi A, 2018 [54]

**Table 2:** Ongoing trials of oligometastatic and oligoprogressive breast cancer patients.

Reference	Setting	Intervention	Radiotherapy Dose/Volumes	Primary Endpoints
CLEAR, Jeong J, NCT03750396	Oligometastatic breast cancer recurrence (>12 months) All site of metastases	Surgery or radiotherapy or radiofrequency on metastasis	Total radiation dose and fractions are various according to metastatic lesions (57–97.5 Gy/6–10 Fraction)	PFS
NRG Oncology, NCT02364557	Limited MBC	SBRT +/− Surgery	Radiosurgery in 1, 3 or 5 fractions (according to discretion of physician)	PFS OS
STEREO-SEIN, NCT02089100	De novo Oligometastatic Breast Cancer, excluding triple negative subtype	SBRT	SBRT with radical intent to all sites of metastases	PFS
MSKCC, NCT03808337	Metastatic NSCLC or TNBC	SBRT concurrently to systemic therapy	SBRT with a minimum BED of 48 Gy to all sites	PFS
NCI, NCT00182793	Stage IIIb-IV BC	RT on primary site or on site of metastasis (oligometastatic), High-dose chemotherapy, autologous stem cells transplant	Tomotherapy on site of disease with standard fractionation	5-year Relapse-Free-Survival 5-year Overall, Survival-Rate
CIMER, NCT04220476	Oligometastatic, Luminal BC	SBRT (Immune-SBRT every 48 h)	SBRT every 48 h, to all sites of metastases 50GY in 5 fractions	ORR PFS OS
MSKCC NCT03808662	Oligoprogressive NSCLC or TNBC	SBRT	SBRT 9–10 Gy × 3 or 10 Gy × 5 fractions given every other day to all sites	PFS
